# The implementation of cardiac arrest treatment recommendations in English acute NHS trusts: a national survey

**DOI:** 10.1136/postgradmedj-2016-134732

**Published:** 2017-04-25

**Authors:** James Carberry, Keith Couper, Joyce Yeung

**Affiliations:** 1 University of Birmingham, Edgbaston, Birmingham, UK; 2 Warwick Medical School, University of Warwick, Coventry, UK; 3 Academic Department of Anaesthesia, Critical Care, Pain and Resuscitation, Heart of England NHS Foundation Trust, Birmingham, UK

**Keywords:** Cardiac Arrest, cardiopulmonary resuscitation, Knowledge Translation

## Abstract

**Purpose of the study:**

There are approximately 35 000 in-hospital cardiac arrests in the UK each year. Successful resuscitation requires integration of the medical science, training and education of clinicians and implementation of best practice in the clinical setting. In 2015, the International Liaison Committee on Resuscitation (ILCOR) published its latest resuscitation treatment recommendations. It is currently unknown the extent to which these treatment recommendations have been successfully implemented in practice in English NHS acute hospital trusts.

**Methods:**

We conducted an electronic survey of English acute NHS trusts to assess the implementation of key ILCOR resuscitation treatment recommendations in relation to in-hospital cardiac arrest practice at English NHS acute hospital trusts.

**Results:**

Of 137 eligible trusts, 73 responded to the survey (response rate 53.3%). The survey identified significant variation in the implementation of ILCOR recommendations. In particular, the use of waveform capnography (n=33, 45.2%) and ultrasound (n=29, 39.7%) was often reported to be available only in specialist areas. Post-resuscitation debriefing occurs following every in-hospital cardiac arrest in few trusts (5.5%, n=4), despite a strong ILCOR recommendation. In contrast, participation in a range of quality improvement strategies such as the National Cardiac Arrest Audit (90.4%, n=66) and resuscitation equipment provision/audit (91.8%, n=67) were high. Financial restrictions were identified by 65.8% (n=48) as the main barrier to guideline implementation.

**Conclusion:**

Our survey found that ILCOR treatment recommendations had not been fully implemented in most English NHS acute hospital trusts. Further work is required to better understand barriers to implementation.

## Introduction

In-hospital cardiac arrest (IHCA) is a true medical emergency, in which the delivery of time-critical high-quality care is challenging. In the UK there are 1.5 IHCAs per 1000 hospital admissions, with just 18.4% of patients surviving to hospital discharge.^1^

In the UK, cardiac arrest treatment guidelines are developed by the Resuscitation Council (UK).^2^ These guidelines are based on treatment recommendations developed by the International Liaison Committee on Resuscitation (ILCOR) which are reviewed and published every 5 years in the journals *Circulation* and *Resuscitation*.^2–4^ The latest ILCOR review process, which concluded in 2015, involved the completion of 169 systematic reviews across seven domains (adult basic life support; adult advanced life support; acute coronary syndrome; paediatric basic life support; neonatal resuscitation; education, implementation and teams; first aid).^3^ These reviews were conducted using the robust GRADE (Grading of Recommendations Assessment, Development and Evaluation) methodology, which culminated in teams of resuscitation experts making a recommendation or suggestion for or against an intervention.^5 6^

The Resuscitation Council (UK) guidelines and the ILCOR treatment recommendations on science are broadly similar, although there are subtle differences due to the purpose of the two documents.^2 3^ The key difference is that the ILCOR treatment recommendations are broader in scope and cover all elements of resuscitation science. This includes detailed recommendations on acute coronary syndrome and training delivery, which are not covered in detail in the Resuscitation Council (UK) guidelines. This approach ensures that the Resuscitation Council (UK) guidelines are tailored to the UK context.

The cardiac arrest formula for survival describes three components that are essential to optimise survival following cardiac arrest—namely, medical science, education and implementation.^7^ This concept of a formula describes survival as a function of how well each of the three components exist in practice. As such, for optimal survival, medical science must be developed, taught and implemented in practice. However, it is presently unknown how well and quickly resuscitation treatment recommendations are implemented by English acute hospital trusts. The publication of the latest ILCOR treatment recommendations in October 2015 provides a timely opportunity to review this implementation.^3^

## Methods

Between February and March 2016 we conducted a national electronic survey of all English acute NHS trusts to assess their implementation of the 2015 ILCOR treatment recommendations. Baseline data on key NHS trust characteristics, such as the number of hospitals and beds, were collected from Department of Health data. The electronic survey was hosted by SurveyMonkey (Palo Alto, California, USA).

The main questionnaire comprised 28 questions that covered the implementation of key treatment recommendations across four ILCOR domains: adult basic life support (BLS); adult advanced life support (ALS); acute coronary syndrome (ACS); and education, implementation and teams (EIT).^8–11^ To develop the survey, the study authors reviewed the 2015 ILCOR treatment recommendations and developed a pool of questions relevant to IHCA practice. The authors then ranked specific topic areas based on their perceived importance in terms of relevance to UK hospital setting and uncertainty of practice. As many questions considered important were selected as possible, while attempting to ensure that the survey was simple to navigate and quick to complete in order to maximise the response rate. The survey was piloted with resuscitation officers from two NHS trusts to ensure that the questions were valid and would be interpreted correctly by the intended audience. Minor amendments to the survey were made following feedback.

### Inclusion/exclusion criteria

NHS trusts in England were eligible to participate in the survey if they were an NHS hospital trust that provided general acute adult secondary care. NHS trusts were not eligible if they were a specialist trust providing only paediatric care, women’s care, mental healthcare or tertiary services.

### Data collection

Eligible trusts were identified using a Department of Health list of NHS trusts.^12^ Resuscitation officers are specialist practitioners that typically have a combined training, clinical service, quality assurance and research role in the area of resuscitation.^13^ As such, trust resuscitation officers were identified as the most appropriate person to complete the survey on behalf of their respective NHS trust.

A three-stage contact approach was adopted. First, an initial email was sent to the resuscitation officer at each eligible trust inviting them to complete the survey on behalf of the trust. A second reminder email was sent a week later if there was no reply. Finally, in the event of further non-response, the resuscitation officer was contacted by telephone.

### Data analysis

Data were downloaded and imported into Microsoft Excel (Microsoft Corporation, Washington, USA) to facilitate a descriptive analysis. Categorical data are reported as frequency and percentage. Continuous data were assessed for normality. Normally distributed continuous data are reported as mean and 95% CI. Non-normally distributed data are reported as median and IQR.

## Results

**Figure 1:**
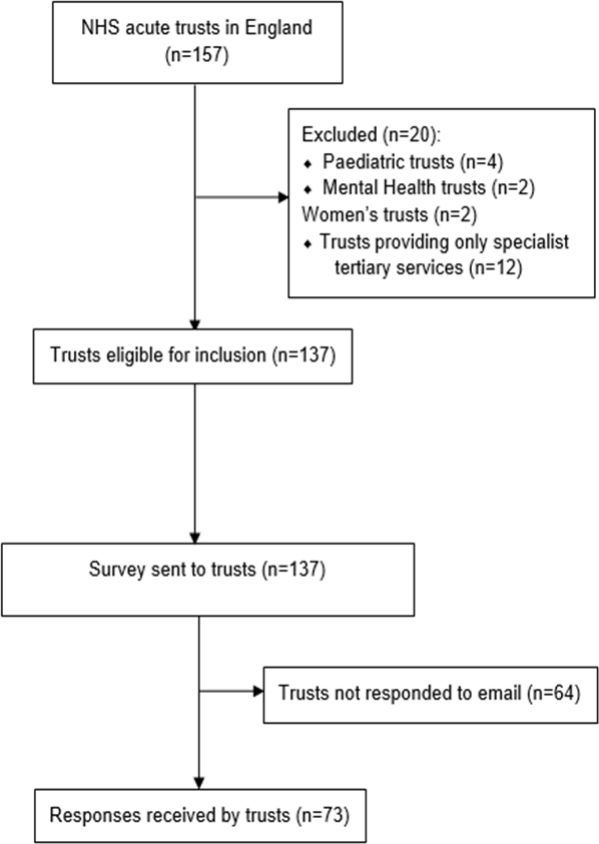
Progress through the study from identification of target population to completion of survey (n=number of trusts).

A total of 157 acute NHS trusts were identified in England, of which 20 were deemed not eligible to participate in the study due to being a mental health trust (n=2), paediatric care only trust (n=4), women’s care only trust (n=2) or a trust providing only specialist tertiary services (n=12) (figure 1). Of the 137 eligible trusts, the survey was completed by 73 trusts (response rate 53.3%).

### Characteristics of participating hospitals

Most responding trusts managed more than one hospital (n=45, 61.9%), with a median number of hospital beds of 660 (IQR 450–950). The median numbers of whole time equivalent (WTE) junior and senior resuscitation officers funded by each trust was 1 (IQR 0–2.1) and 2 (IQR 1–3), respectively. Most trusts (n=32, 43.8%) reported a cardiac arrest incidence of 1–1.99 IHCAs per 1000 admissions, with 16 (21.9%) respondents unsure of the IHCA incidence at their trusts. There were small differences between responding and non-responding trusts in relation to the number of hospitals managed and number of beds (table 1).

**Table 1:** Characteristics of participating and non-participating hospitals

	Responding hospitals (n=73)	Non-responding hospitals (n=64)
Hospitals managed by trust, n (%)		
1	28 (38.4)	17 (26.6)
2	22 (30.1)	18 (28.1)
3	12 (16.4)	13 (20.3)
≥4	11 (15.1)	16 (25.0)
Number of beds, median (IQR)	660 (450–950)	769 (580–1027)
Number of WTE resuscitation officers, median (IQR)		
Junior (band 5/6)	1 (0–2.1)	
Senior (band 7/8)	2 (1–3)	
Cardiac arrests per 1000 admissions, n (%)		
0–0.99	7 (9.6)	
1–1.99	32 (43.8)	
2–2.99	10 (13.7)	
≥3	8 (11.0)	
Unknown	16 (21.9)	

WTE, whole time equivalent.

Tables 2–4 summarise the implementation of guidelines in relation to devices and treatments; pre-briefing and debriefing; BLS and ALS training provision. Each table incorporates the relevant ILCOR treatment recommendation for each question.^8–11^

**Table 2:** Devices and treatments

			Respondents (n=73)	ILCOR recommendation 2015
Mechanical chest compression device				
	Hospital has mechanical device, n (%)		49 (67.1)	‘We suggest against the routine use of automated mechanical chest compression devices but suggest they are a reasonable alternative to use in situations where sustained high-quality manual chest compressions are impractical or compromise provider safety’ (Weak recommendation)^10^
	Devices currently used by hospitals*, n (%)			
		Autopulse (ZOLL Medical Corporation, Chelmsford, MA, USA)	19 (38.8)	
		LUCAS (Physio-Control Inc/Jolife AB, Lund, Sweden)	35 (71.4)	
	Indications for use of a mechanical device*, n (%)			
		Routinely used at all cardiac arrests	1 (2.0)	
		ED cardiac arrests	21 (42.9)	
		Cardiac catheter laboratory cardiac arrests	26 (53.1)	
		Patients in cardiac arrest requiring transfer	10 (20.4)	
		Cardiac arrest in CT scanner	1 (2.0)	
		Prolonged cardiac arrest	32 (65.3)	
		Mechanical device not routinely available	2 (4.1)	
Waveform gapnography				‘We recommend using waveform capnography to confirm and continuously monitor the position of a tracheal tube during CPR in addition to clinical assessment’ (strong recommendation)^10^
	Hospital routinely uses waveform capnography during cardiac arrest events, n (%)			
		Yes – use at all cardiac arrests	26 (35.6)	
		Yes – where available/specific locations (eg, ED, ITU only)	33 (45.2)	
		No	14 (19.2)	
Ultrasound				‘We suggest that if cardiac ultrasound can be performed without interfering with standard ACLS protocol, it may be considered as an additional diagnostic tool to identify potentially reversible causes’ (weak recommendation)^10^
	Ultrasound used during CPR, n (%)			
		Yes – routinely available on all wards	3 (4.1)	
		Yes – if skilled personnel available	34 (46.6)	
		Yes – restricted to ED/ITU	29 (39.7)	
		No	7 (9.6)	
Extracorporeal membrane oxygenation				‘We suggest ECPR is a reasonable rescue therapy for selected patients with cardiac arrest when initial conventional CPR is failing in settings where this can be implemented’ (weak recommendation)^10^
	Hospital has access to extracorporeal membrane oxygenation for cardiac arrest patients, n (%)		8 (11.0)	
CPR prompt/feedback devices				‘We suggest the use of real-time audiovisual feedback and prompt devices during CPR in clinical practice as part of a comprehensive system for care for cardiac arrest’ (weak recommendation)^8^ ‘We suggest against the use of real-time audiovisual feedback and prompt devices in isolation (ie, not part of a comprehensive system of care)’ (weak recommendation)^8^
	CPR prompt/feedback devices used by hospitals during CPR, n (%)			
		Metronome	10 (13.7)	
		Accelerometer-based device	12 (16.4)	
		Other device	2 (2.7)	
		CPR prompt/feedback devices not used routinely during CPR	54 (74.0)	
Patients where primary percutaneous coronary intervention is considered post-arrest, n (%)				
		STEMI	50 (68.5)	‘We recommend emergency cardiac catheterisation laboratory evaluation in comparison with cardiac catheterisation later in the hospital stay or no catheterisation in select adult patients with ROSC after OHCA of suspected cardiac origin with ST elevation on ECG’ (strong recommendation)^7^ ‘We suggest emergency cardiac catheterisation laboratory evaluation in comparison with cardiac catheterisation later in the hospital stay or no catheterisation in select adult patients who are comatose with ROSC after OHCA of suspected cardiac origin without ST elevation on ECG’ (weak recommendation)^7^
		Other (not STEMI) with ECG changes and likely cardiac cause	29 (39.7)	
		All patients with likely cardiac cause	26 (35.6)	
		PCI not available – thrombolysis considered for STEMI	1 (1.4)	
		No patients	0 (0)	
		Unsure	6 (8.2)	

*Multiple answers allowed.

ILCOR, International Liaison Committee on Resuscitation; OHCA, Out of Hospital Cardiac Arrest; ROSC, Return of Spontaneous Circulation; STEMI, ST-elevation myocardial infarction.

**Table 3:** Debriefing and quality improvement

			Respondents (n=73)	ILCOR recommendation 2015
Debriefing				
	Trust runs formal programme for staff feedback/debriefing on their performance following IHCA, n (%)			
		Yes – for every arrest	4 (5.5)	We recommend data-driven, performance-focused debriefing of rescuers after IHCA in both adults and children’ (strong recommendation)
		Yes – for some arrests	36 (49.3)	
		Unsure	2 (2.7)	
		No	31 (42.5)	
	Types of feedback/debriefing offered to staff, n (%)			
		Hot debrief (without CPR quality data)	33 (82.5)	
		Hot debrief (with CPR quality data)	3 (7.5)	
		Cold debrief (without CPR quality data)	26 (65.0)	
		Cold debrief (with CPR quality data)	7 (17.5)	
		Written feedback (without CPR quality data)	5 (12.5)	
		Written feedback (with CPR quality data)	2 (5.0)	
	Focus of debrief (1, not important to 5, key focus)			
		Education/quality of care issues, median (IQR)	4 (4—5)	
		Psychological/emotional issues, median (IQR)	4 (4—5)	
Quality Improvement				
	Quality improvement strategies in use at hospitals, n (%)			
		Participation in National Cardiac Arrest Audit (NCAA)	66 (90.4)	‘We suggest the use of performance measurement and quality improvement initiatives in organisations that treat cardiac arrest’ (weak recommendation)
		Patient outcome review	49 (67.1)	
		CPR quality review	21 (28.8)	
		Rolling CPR refreshers	49 (67.1)	
		In situ cardiac arrest simulation	52 (71.2)	
		Real-time CPR feedback	21 (28.8)	
		Debriefing	41 (56.2)	
		DNAR documentation audit/review	66 (90.4)	
		Incident reporting review	57 (78.1)	
		Resuscitation equipment provision/audit	67 (91.8)	

DNAR, Do Not Attempt Resuscitation; ILCOR, International Liaison Committee on Resuscitation.

**Table 4:** Basic life support and advanced life support training provision

			Respondents (n=73)	ILCOR recommendation 2015
BLS training				
	Methods of delivery used for BLS teaching, n (%)			
		Self-instruction (eg, DVD, e-learning)	1 (1.4)	‘We suggest that video and/or computer-based self-instruction with synchronous or asynchronous hands-on practice may be an effective alternative to instructor-led courses’ (Weak recommendation)
		Instructor-led	51 (69.9)	
		Both	21 (28.8)	
	CPR prompt/feedback devices used during BLS training, n (%)			
		Metronome	19 (26.0)	‘We suggest the use of feedback devices that provide directive feedback on compression rate, depth, release, and hand position during training’ (Weak recommendation) ‘If feedback devices are not available, we suggest the use of tonal guidance (examples include music or metronome) during training to improve compression rate only’ (Weak recommendation)
		Real-time feedback devices	35 (47.9)	
		Manikin-based feedback	29 (39.8)	
		Prompt/feedback device not used in BLS training	22 (30.1)	
	Use of high-fidelity manikins during BLS training, n (%)			
		Yes	13 (17.8)	‘We suggest the use of high-fidelity manikins when training centres/organisations have the infrastructure, trained personnel, and resources to maintain the programme’ (Weak recommendation)
		When available	11 (15.1)	
		No	49 (67.1)	
ALS training				
	CPR prompt/feedback devices used during ALS training, n (%)			
		Metronome	16 (21.9)	‘We suggest the use of feedback devices that provide directive feedback on compression rate, depth, release, and hand position during training’ (Weak recommendation) ‘If feedback devices are not available, we suggest the use of tonal guidance (examples include music or metronome) during training to improve compression rate only’ (Weak recommendation)
		Real-time feedback devices	36 (49.3)	
		Manikin-based feedback	33 (45.2)	
		Prompt/feedback device not used in ALS training	12 (16.4)	
	Use of high-fidelity manikins during ALS training, n (%)			
		Yes routinely	35 (47.9)	‘We suggest the use of high-fidelity manikins when training centres/organisations have the infrastructure, trained personnel, and resources to maintain the programme’ (Weak recommendation) ‘If high-fidelity manikins are not available, we suggest that the use of low-fidelity manikins is acceptable for standard ALS training in an educational setting’ (Weak recommendation)
		Used when available	9 (12.3)	
		Not used routinely	29 (39.7)	

ALS, adult advanced life support; BLS, basic life support; ILCOR, International Liaison Committee on Resuscitation.

### Devices and treatment during cardiac arrests in the clinical setting

Respondents were asked about the availability of medical devices that were cited by the international resuscitation guidelines (table 2). Mechanical chest compression devices that could deliver consistent high quality chest compressions were owned by 67.1% of trusts surveyed (n=49), with common indications for use reported to be prolonged cardiac arrests (n=32, 65.3%), cardiac catheter laboratory cardiac arrests (n=26, 53.1%) and emergency department cardiac arrests (n=21, 42.9%). Only one trust reported that devices are used routinely at all cardiac arrests (n=1, 2.0%).

Ultrasound (n=66, 90.4%) and waveform capnography (n=59, 80.8%) were reported to be available at the majority of trusts, but their use was not routine during cardiac arrests. In particular, waveform capnography was often available in specialist clinical areas only, with only a minority of trusts reporting its availability at all cardiac arrests (n=26, 35.6%). Similarly, ultrasound was not routinely used with only three (4.1%) trusts reporting it routinely available at all cardiac arrests.

Most trusts (n=54, 74.0%) did not use a CPR prompt or feedback device to monitor quality of CPR during IHCAs. In trusts where a device was used, accelerometer-based devices were the most commonly used devices, which were used in 12 (16.4%) trusts.

In terms of treatment for cardiac arrests, access to the use of extracorporeal membrane oxygenation for patients in cardiac arrest was very limited and only available in eight (11.0%) trusts.

In most trusts (68.5%, n=50), patients with ST-elevation myocardial infarction (STEMI) were considered for primary percutaneous coronary intervention (pPCI), either at their own centre or referred for consideration elsewhere. Patients without STEMI but who had ECG changes and a likely cardiac cause of the arrest were considered for pPCI at 29 (39.7%) trusts. There were a similar number of trusts (n=26, 35.6%) where all patients with a likely cardiac cause of the arrest were considered for pPCI.

### Debriefing and quality improvement

Post cardiac arrest debriefing can be used to educate staff and improve overall CPR quality. Just over half of trusts (n=40, 54.7%) reported that debriefing is provided following cardiac arrest (table 3). In trusts that provided debriefing, CPR quality data were not routinely used, with most commonly used modalities being an immediate (hot) debrief without CPR quality data (n=33, 82.5%) and a delayed (cold) debrief without CPR quality data (n=26, 65.0%). It is possible that CPR quality data were simply not available due to the lack of feedback devices used in cardiac arrests. Both educational (quality of care) and psychological (emotional) issues were considered important focuses of debriefing by trusts.

The vast majority of responding trusts were engaged in quality improvement interventions with more than 90% participating in the National Cardiac Arrest Audit (NCAA). This included resuscitation equipment audits (n=67, 91.8%), participation in the NCCA (n=66, 90.4%) and audit of Do Not Attempt Cardiopulmonary Resuscitation (DNACPR) documentation (n=66, 90.4%).

### Basic life support (BLS) and advanced life support (ALS) training provision

Clinical staff working in the NHS require regular training in resuscitation skills including BLS and ALS. In responding trusts, most delivered only instructor-led BLS training (table 4, n=51, 69.9%). Some trusts (n=21, 28.8%) reported using other methods such as a combination of instructor-led and self-instruction teaching. Feedback devices were more popular with training, with 51 trusts (69.9%) reporting the use of CPR prompt/feedback devices during BLS training and popular devices included real-time feedback devices (n=35, 47.9%), manikin-based feedback (n=29, 39.8%) and metronomes (n=19, 26.0%). A greater proportion of trusts used CPR prompt/feedback devices during ALS training, with the most commonly used devices being real-time feedback devices (n=36, 49.3%), manikin-based feedback (n=33, 45.2%) and metronome devices (n=16, 21.9%).

High-fidelity manikins are rarely used during BLS training by responding trusts (always n=13, 17.8%; when available n=11, 15.1%). High-fidelity manikins are used more commonly during ALS training, such that 35 (47.9%) trusts use them regularly during ALS training and 9 (12.3%) use them when available.

### Perceived barriers to implementation of clinical guidelines

In response to what were the perceived barriers to implementing clinical guidelines, the most frequently reported barriers were financial factors (n=48, 65.8%) and levels of staffing (30.1%, n=22). A minority of trusts reported time (n=5, 6.8%) and the low quality of the evidence underpinning many recommendations (n=2, 2.7%) as barriers. Eight trusts (11.0%) reported no barriers to implementation.

## Discussion

In this survey of the implementation of ILCOR treatment recommendations for in-hospital cardiac arrest at English acute NHS trusts, we found evidence of marked variability in practice. These disparities did not appear to correlate to the strength of treatment recommendation as summarised in tables 2–4. We found evidence that recommended interventions, such as waveform capnography and ultrasound, are often available only to select patient groups or have yet to be implemented.

The limited compliance in some areas of our survey may be attributable to the short time-span between undertaking our survey and release of the ILCOR treatment recommendations and Resuscitation Council (UK) guidelines. While this was not identified as a barrier to implementation by any trust, it should be recognised that this short duration may not have been sufficient for effective implementation, particularly where additional funding or staffing is required. From this perspective, our survey may provide a useful benchmark to assess implementation rates in the future.

Debriefing provides an interesting example where implementation has not fully reflected the ILCOR treatment recommendation. The implementation of cardiac arrest debriefing is associated with improvements in CPR quality and patient outcome.^14–16^ Effective debriefing is reliant on the availability of objective performance data, such as defibrillator CPR quality downloads or video-recordings.^17^ This is reflected in the ILCOR recommendation that debriefing should be ‘data-driven’ and ‘performance-focused’.^10^ However, our survey found that, while many trusts undertook debriefing, although perhaps on an ad hoc basis, this process was rarely supported by CPR quality data such that the effectiveness of such debriefing may be limited.

To our knowledge, this is the first English survey that has covered a broad range of IHCA practices. Previous surveys of resuscitation have focused on specific areas such as waveform capnography and post-resuscitation care.^18–21^ These surveys have similarly identified variability in the implementation of guidelines.

There have been two previous surveys of a broad range of IHCA practices conducted in other countries.^22 23^ Edelson *et al* surveyed practice across 439 North American hospitals (response rate 44%).^22^ The survey reported infrequent implementation in hospitals of capnography (25%), real-time audiovisual feedback devices (4%) and debriefing (34%). The survey by Tirkkonen *et al* of 51 Finnish hospitals (response rate 93%) focused on the characteristics of hospital medical emergency and cardiac arrest teams but also reported that, in hospitals with intensive care units, a single hospital (3.4%) debriefed the team following cardiac arrests and most (72.4%) used real-time audiovisual feedback technology.^23^ As such, in combination with our survey, these surveys highlight marked variability in in-hospital resuscitation practice both within nations and between nations.

The implementation of evidence-based guidelines in practice is challenging, and there is a need for further work to examine the best approaches to successful implementation in the context of resuscitation.^24^ Our finding that finance and staffing levels were recognised as the key barriers to implementation is unsurprising, particularly in the publicly funded UK National Health Service. However, in contrast to previous surveys, the strength of evidence supporting ILCOR treatment recommendations was not considered a significant barrier to implementation.^20 21^

The key strength of our study is that it is, to our knowledge, the first English survey of in-hospital practices that covers a range of practice areas. It does, however, have some limitations. First, the response rate was 53.3%. While key demographics (number of beds and hospitals) were broadly similar between responding and non-responding trusts, such a response creates a risk of selection bias. Poor response rates are common in surveys of health professionals, with surveys often reporting response rates similar to that achieved in this study.^25^ We sought to maximise the response rate by limiting the survey length and making multiple contact attempts. Second, we collected data through an electronic survey that was completed by resuscitation officers. Answers were likely based on organisational policy, which may not necessarily reflect actual practice.

## Conclusion

Our survey highlighted variability in IHCA practice across NHS hospitals in England. Many trusts are yet to implement key ILCOR treatment recommendations, with key barriers being financial factors and staffing levels. Our results highlight the need to optimise the final component (‘implementation’) in the cardiac arrest formula for survival to maximise the likelihood of survival for NHS patients.

**Main messages**

- International Liaison Committee on Resuscitation treatment recommendations are yet to be fully implemented across most acute adult NHS hospital trusts
- The key barriers to implementation of treatment recommendations are financial and staffing
- There is variability in resuscitation practice for adult in-hospital cardiac arrest across acute adult NHS hospital trusts

**Current research questions**

- How can barriers to implementation of treatment recommendations be addressed?
- What factors influence the prioritisation of implementing some treatment recommendations over others?
- What is the impact of variability in resuscitation practices on patient outcome?
